# Predicting students’ performance on geometric problem-solving tasks: the roles of cognitive reflection, fluid intelligence, and mathematical beliefs

**DOI:** 10.3389/fpsyg.2026.1809096

**Published:** 2026-06-09

**Authors:** Alicia Rubio-Sánchez, Isabel Gómez-Veiga, Inés M. Gómez-Chacón

**Affiliations:** 1 Escuela Internacional de Doctorado, Universidad Nacional de Educación a Distancia (UNED), Madrid, Spain; 2 CUNEF Universidad, Madrid, Spain; 3 Departamento de Psicología Evolutiva y de la Educación, Facultad de Psicología, Universidad Nacional de Educación a Distancia (UNED), Madrid, Spain; 4 Instituto de Matemática Interdisciplinar, Universidad Complutense de Madrid (UCM), Madrid, Spain

**Keywords:** beliefs, fluid intelligence, gender, geometry, mathematics, reasoning

## Abstract

**Introduction:**

Recent perspectives in mathematics education highlight the importance of integrating cognitive and affective dimensions in understanding students’ reasoning. Accordingly, the present study investigates the interrelations of performance on geometric problem-solving and general cognitive skills, fluid intelligence, cognitive reflection, and mathematics-related beliefs.

**Methods:**

The study explored differences in geometric problem-solving performance by gender and academic year, as well as the predictive contribution of cognitive, mathematics-belief, and mathematics achievement variables. A total of 303 secondary school students (years 8–11) completed several measures assessing geometric problem-solving, cognitive reflection, fluid intelligence, and mathematics-related beliefs; prior mathematics achievement was obtained from official school grades. Correlational and hierarchical regression analyses were conducted to examine predictive relationships, while moderation and mediation analyses were performed to test interaction effects and indirect effects, respectively.

**Results:**

Results indicated significant differences in geometric problem-solving performance across academic years, with no significant gender differences. Cognitive reflection, fluid intelligence, and mathematics achievement emerged as significant predictors of performance on the geometric problem-solving task, while mathematics-related beliefs were significantly correlated with geometry performance but did not explain additional variance once cognitive and achievement variables were considered. Mediation analyses did not support significant indirect effects through mathematics-related beliefs, and moderation analyses did not show significant gender- or grade-level differences in the associations between cognitive predictors and geometry performance.

**Discussion:**

These findings highlight the relevance of reasoning processes in adolescents’ geometric problem-solving and provide further evidence of the role of cognitive and belief-related factors in its development.

## 1 Introduction

Geometry education poses challenges for both teaching and learning as reflected in the literature (Jablonski and Ludwig, 2023; Jones and Tzekaki, 2016; Mammarella et al., 2017; Sinclair et al., 2016). Geometry has gained prominence in school curricula not only as an autonomous domain of mathematics but also due to its broader contribution to mathematical learning. The nature of its concepts, together with the geometric reasoning requirements, involved in establishing connections across mental representations and linking them to formal mathematical principles make this domain particularly demanding for students. In this context, students’ geometric problem-solving involves reasoning processes that are supported by both cognitive and affective factors. Within the cognitive domain, cognitive reflection and fluid intelligence have been identified as key predictors of students’ engagement in analytical and deliberate thinking (Evans and Stanovich, 2013; Frederick, 2005; Toplak et al., 2011), in line with dual-process theories of reasoning (Cattell, 1963; De Neys, 2006; Primi et al., 2016; Roth et al., 2015). The role of dual process theories in mathematical education is a topic of interest, and some studies have linked it with mathematical learning (Borodin, 2016) and mathematical reasoning (Gómez-Chacón et al., 2014; Leron and Hazzan, 2009). In mathematical contexts, and particularly in geometry, effective problem-solving often requires shifting from intuitive responses to more reflective processes in order to avoid superficial judgments and reach abstract, logically valid conclusions. Likewise, the subject metacognitively evaluates that conflict and decides whether to continue reasoning or accept the initial intuition. In this metacognition dimension, the role of confidence and people’s evaluation of their own responses is especially significant (De Neys, 2006, De Neys, 2018; Thompson et al., 2011).

Drawing on Duval’s theoretical framework, geometric reasoning is here situated within problem-solving tasks, where the transformation and representation of geometric figures constitute essential cognitive operations. The combination of both approaches (dual-process theories and Duval’s theory of semiotic representations) allows us to explain visual biases in geometry reasoning, persistent errors between changes of representation register from algebraic to geometric and contradictions between intuition and formal reasoning.

In this study, using different measurement instruments (performance on geometric problem-solving, cognitive reflection, and fluid intelligence), we have sought to understand forms of reasoning and capture complementary dimensions of individuals’ cognitive activity. Likewise, beyond the cognitive dimension, the affective dimension also plays a crucial role. Students’ beliefs about mathematics can shape their approach to reasoning and problem-solving, while their academic achievement in mathematics provides a developmental indicator of their broader mathematical competence. Taking together, these variables allow for a more comprehensive understanding of the factors linked to adolescents’ performance on geometric problem-solving.

### 1.1 Geometric reasoning

Geometric reasoning is conceived in this study as a complex cognitive process in which individuals mentally represent, transform, and operate on geometric figures and spatial relationships in the context of problem-solving. It involves coordinating visual, discursive, and symbolic modalities in order to analyze properties, identify patterns, formulate conjectures, and engage in analytical reasoning processes. In this sense, reasoning in geometry goes beyond visualization and involves the integration of multiple semiotic registers and modes of apprehension that allow learners to engage meaningfully with geometric structures and transformations (Duval, 1995a, 1995b, 1998, 2006).

Among the various theoretical frameworks that explore students’ geometrical reasoning, Duval’s theory of semiotic representations (Duval, 2006) is particularly influential. Duval identifies three core cognitive processes involved in geometrical reasoning: visualization, construction, and reasoning. Visualization refers to the use of visual representations to interpret or heuristically explore complex geometrical situations. In Duval’s sense, construction denotes the operative production and manipulation of geometric configurations, including both instrumental (like the use of ruler-and-compass or dynamic-geometry tools) or mental configuration (like anticipating the outcomes of figural operations such as rotating or translating). Reasoning, in turn, is defined as the discursive elaboration of statements that are built upon known concepts with the aim of justifying, proving, or explaining geometrical relationships (Duval, 1998, 2006). This framework highlights that progress in geometry depends on students’ ability to coordinate representations, rather than relying solely on intuition or imagery. Visualization is thus only one dimension of reasoning, integrated into a broader representational account that explains how students construct meaning and advance toward abstraction.

In line with this perspective, recent studies in the field have further emphasized the central role of representations in geometry learning and explored the cognitive structure of the geometrical figure apprehension dimensions (operative, discursive, and perceptual) in three grades of secondary school students (Gagatsis et al., 2022; Michael-Chrysanthou et al., 2024). In Jablonski and Ludwig (2023) was presented a literature review on theory and practice in geometry identifies representational practices like drawing, sketching, and the use of inscriptions as central in the current research in the field, that also considers as relevant reasoning, proof, digital tools, and the role of language and drawings in mathematical activity. Based on their systematic review of research on dynamic geometry, Weigand et al. (2025) highlight how such tools mediate students’ engagement with drawing practices, problem-solving approaches, and reasoning processes, particularly in relation to formulating conjectures and exploring geometric relationships. Similarly, Castro et al. (2022) show that research conducted within Latin American community has concentrated heavily on Euclidean and spatial geometry, often in the form of theoretical essays, while underscoring the need for closer connections to pedagogical practice and assessment. This sustained attention to representational activity reflects an increasing recognition that reasoning in geometry is inseparable from the ways learners construct, manipulate, and interpret representations.

These perspectives align with empirical findings that illustrate how geometric reasoning supports students’ conceptual development and problem-solving in mathematics. Recent contributions, such as Kuzle (2022), maintain that geometric reasoning permeates all domains of mathematics, given its foundational role in the processes of perceiving, analyzing, and encoding visual information. Her work suggests that sustained engagement with geometric content not only facilitates the development of basic cognitive skills but also supports distinct forms of mathematical ways of thinking that are instrumental for the student’s overall understanding.

In addition, Juman et al. (2022) argue that in the process of learning geometry, students engage with multiple representations, such as virtual manipulatives, symbolic notation, and verbal explanations, which collectively support their construction of mathematical concepts and to develop critical thinking. Similarly, Michael-Chrysanthou et al. (2024) argue that the development of geometric reasoning is essential, particularly in secondary education, as it fosters students´ ability to visualize, formulate conjectures, and construct logically grounded arguments based on geometric principles. By using figures, students are able to understand, analyze, and make logical inferences about shapes, spatial relationships, and geometric structures. Collectively, these studies support the view that geometric reasoning is not a peripheral skill but rather a fundamental mode of mathematical thought, grounded in the coordination of representational resources.

Regarding these contributions, this study adopts Duval’s framework as the primary lens through which geometric reasoning is conceptualized. We consider in this study that geometric reasoning does not occur in isolation but interacts with broader general cognitive skills, such as cognitive reflection and fluid intelligence, and with affective dimensions, including students’ beliefs about mathematics and their academic achievement. Within this theoretical framework, beliefs and self-efficacy beliefs are considered affective variables that may shape students’ engagement with demanding geometric tasks requiring the coordination of visual, textual, and formal information (Street et al., 2024). The following section addresses these variables and reviews the evidence regarding their role and influence in adolescents’ performance on geometric solving tasks.

### 1.2 General cognitive variables: fluid intelligence and cognitive reflection

Fluid intelligence (Gf) is defined as the ability to think abstractly, reason logically, and solve novel problems (Cattell, 1963). It is widely considered the best single predictor of general academic achievement (Deary et al., 2007), particularly in science and mathematics (Lin et al., 2021; Peng et al., 2019; Roth et al., 2015). Peng et al.’s (2019) meta-analysis concluded that Gf shows moderate but reciprocal relations with mathematics, with stronger associations when tasks involve complex mathematics and composite nonverbal reasoning. Likewise, authors such as Kyttälä and Lehto (2008) have pointed out some factors underlying mathematical performance, highlighting the role of visuospatial working memory and non-verbal intelligence.

Although fewer studies have specifically addressed geometry specifically, the results show that these relationships can be extended in this field. Giofrè et al. (2014), Greiff and Neubert (2014), and Mammarella et al. (2017) proposed models in which intelligence explained a significant proportion of variance in students’ achievement in geometry, reinforcing the importance of Gf in this domain.

The relevance of fluid intelligence in geometry is consistent with the cognitive demands described by dual-process theories of reasoning, particularly those involving conscious, controlled processes that rely on normative, logical, and analytical thinking. Because these forms of reasoning are cognitively demanding, they depend heavily on resources such as working memory and fluid intelligence. From this perspective, fluid intelligence (Gf) can be understood as a core cognitive resource that supports the controlled reasoning and representational coordination required for geometric problem-solving.

Cognitive reflection is a regulation process that represents pivotal process within this framework. Defined as the ability to resist an intuitive but incorrect answer coming from System 1 in favor of a more deliberate and reasoned response (from System 2), cognitive reflection is most frequently measured using the Cognitive Reflection Test (CRT; Frederick, 2005). Solving CRT problems requires students to override initial System 1 responses and engage reflective System 2 processes. This test has been widely used to explore judgment and decision-making (Sinayev and Peters, 2015; Toplak et al., 2014), logical thinking (Sánchez and Slisko-Ignjatov, 2020; Szaszi et al., 2017), mathematical reasoning (Primi et al., 2018), and both scientific and mathematical performance (Doz and Sliško, 2024). Its predictive capacity for mathematics achievement has also been highlighted (Gómez-Chacón et al., 2014; Gómez-Veiga et al., 2018). Moreover, correlations have been found between CRT performance and a range of cognitive abilities, including numerical ability, verbal ability, spatial-mechanical ability, perceptual speed, and numeracy (Gómez-Chacón et al., 2014; Gómez-Veiga et al., 2018; Otero et al., 2021; Sinayev and Peters, 2015; Toplak et al., 2011). In the study by Dolz et al. (2026), the considered predictors, both general cognitive skills (fluid intelligence and cognitive reflection) as well as specific mathematical skills (logical mathematical language and conditional reasoning), have a significant influence on performance. Despite limited empirical attention to the link between CRT and geometric reasoning, this study proposes that cognitive reflection may play a relevant role in supporting students’ ability to reason geometrically, particularly when tasks require the inhibition of intuitive responses in favor of analytical thought (Babai et al., 2015; Stavy and Tirosh, 2000). Geometric problem-solving requires visual analysis, logical deduction, and spatial reasoning. By inhibiting superficial responses and activating reflective processes, students can move beyond immediate visual impressions of figures to recognize deeper properties and relationships. That is, the conscious activation of reflective processes (System 2 reasoning processes) helps the student to deepen their reasoning, thus approaching the so often needed abstract conceptualization of a geometrical problem. In this way, cognitive reflection fosters the abstraction necessary for advanced geometric reasoning, enabling learners to construct more robust and conceptually grounded solutions.

The connection between cognitive skills and mathematics achievement becomes increasingly evident in adolescence, when intelligence and cognitive reflection contribute both to performance on specific tasks and overall mathematical progress. Academic grades in mathematics hence thus constitute an important educational outcome, reflecting the combined effect of cognitive abilities, problem-solving skills, and affective factors. Evidence suggests that the predictive role of fluid intelligence and cognitive reflection becomes particularly significant as mathematical tasks grow more abstract and complex during secondary education (Mammarella et al., 2017; Peng et al., 2019). In geometry, where reasoning depends on the coordination of representations (Duval, 2006), these abilities provide the cognitive foundation that supports the integration of discursive and visual processes in problem-solving.

Taken together, these findings justify the inclusion of fluid intelligence, cognitive reflection, and mathematical achievement as key variables in the present study. Within dual-process theories and representational activity in geometry, these variables illustrate how general cognitive skills contribute both to mathematics achievement in general and to performance on geometric problem-solving. Previous studies have also examined the combined influence of these variables. For example, Gómez-Veiga et al. (2018) found that intelligence and cognitive reflection significantly predicted mathematics achievement, while Passolunghi et al. (2022) reported that fluid intelligence showed both direct and indirect effects on problem-solving performance. Moreover, Gómez-Chacón et al. (2014) showed that students’ beliefs can moderate the relationship between cognitive reflection and mathematical reasoning, suggesting that affective factors shape the effective use of cognitive resources, a point further elaborated in the next section.

These findings reinforce the predictive role of general cognitive skills across mathematical domains, including the domain of geometry. Although such multivariable approaches are less common in geometry specifically, their extension to this field highlights the relevance of investigating how cognitive and affective variables jointly could support students’ geometric problem-solving performance.

### 1.3 Beliefs about mathematics

However, not only cognitive factors are involved in the process of mathematical and geometrical reasoning. The individual’s belief system also plays a key role in it. As many scholars have argued (De Corte et al., 2010; Goldin et al., 2016; Gómez-Chacón et al., 2006; Gómez-Chacón et al., 2014; Schoenfeld, 1985, 1992), adequate students´ beliefs regarding themselves as mathematics learners play a crucial role in mathematical learning, as those beliefs affect mathematics achievement. Some of these authors further assume that beliefs are not held in isolation but rather constitute coherent belief systems. They consider mainly four types, depending on the object where the belief is focused on: beliefs about mathematics, about one-self (self-efficacy), about mathematics teaching, and finally beliefs about the (social) context where the education in mathematics takes place (De Corte et al., 2010; Gómez-Chacón et al., 2006).

In the present study, we explore belief systems, focusing on the self–beliefs and their relationship with performance in mathematics and geometry. Students self-efficacy is the belief in their capability to succeed in a given task, manage situations, and achieve specific goals (Bandura, 1994). According to Pajares (2008) “self-efficacy should not be confused with self-concept”. Self-efficacy beliefs concern an individual’s perceived ability and center on questions of “can,” whereas self-concept beliefs relate to one’s sense of self and focus on questions of “feel.” Self-efficacy beliefs are often considered in relation to emotional directives towards successful achievement.

When focusing on the influence of beliefs over specific fields of mathematics, often the focus is on arithmetic and algebra (Jasani, 2022). Geometry, on the other hand, is a less common topic. However, examples can be found where authors explore, for example, the role of metacognitive processes, like Suliani et al. (2024), who investigated the relationship between mathematical beliefs and metacognitive processes in geometry problem solving in middle school students. They concluded that mathematical beliefs have a significant positive effect on metacognitive knowledge: students with stronger beliefs were more likely to plan, monitor, and evaluate effectively when solving geometry tasks. Concerning self-efficacy beliefs, a consistent body of evidence shows that mathematics self-efficacy relates positively to persistence, strategy use, and achievement during adolescence (Usher and Pajares, 2009; Yang et al., 2024). In geometry specifically, it is also well-known that students with higher self-efficacy demonstrate greater persistence, confidence, and achievement in geometrical tasks, and are more likely to approach the subject with positive attitudes (Yorulmaz and Çilingir Altıner, 2021).

Panaoura (2014) explored the link between self-efficacy and the coordination of representations in geometry and proposed a model showing that students with higher geometry self-efficacy are more willing and able to engage with multiple representational registers (meaning verbal, symbolic, and visual) when solving problems. This capacity supports not only accuracy but also flexibility in reasoning, as learners with stronger self-efficacy tend to move beyond visual intuition to integrate discursive and analytical modes of thinking. From a cognitive perspective, these findings follow the same line as Duval’s (1995a, 2006) framework, heavily sustained on the posture that progress in geometry relies on the coordination of semiotic representations. Hence, self-efficacy operates as both an affective and regulatory mechanism that facilitates the representational activity required for geometric reasoning.

Building on previous empirical and theoretical research, this study examines the performance on geometric solving task through the interplay between reasoning demands, cognitive skills, mathematical experience, and mathematics-related beliefs. In this framework, fluid intelligence, cognitive reflection, mathematics achievement, and belief systems are treated as complementary factors that may support students’ ability to move beyond immediate visual interpretations and coordinate multiple semiotics representations in geometry. Analyzing their relationships and predictive roles may therefore clarify how these variables contribute to students’ performance on geometric problem-solving during secondary education.

### 1.4 Hypotheses

According to the theoretical background reviewed, the following hypotheses guided the work:

The first hypothesis addresses the interrelations among the general cognitive skills (cognitive reflection and fluid intelligence) and students’ performance on geometric problem-solving, mathematics achievement (school grades) and mathematics-related beliefs. Based on prior findings, we expect a pattern of significant positive correlations among these variables, such that higher cognitive reflection and fluid intelligence, higher mathematics achievement, better performance on geometry and more positive mathematical beliefs (Gómez-Chacón et al., 2014; Gómez-Veiga et al., 2018; Panaoura, 2014).

The second hypothesis addresses the unique predictive contribution of cognitive and affective variables to students´ performance on geometry problem-solving, above and beyond grade level. Specifically, we expect cognitive reflection, fluid intelligence, mathematical achievement, and mathematics-related beliefs to show unique positive contribution to students’ performance on geometric problem-solving tasks. Cognitive reflection and fluid intelligence are expected to emerge as significant predictors, as they provide the cognitive foundation for analytical and deliberate reasoning (Evans and Stanovich, 2013; Primi et al., 2013) and spatial reasoning (Primi et al., 2010). Mathematics achievement (grades) is also expected to contribute positively, as an indicator of prior knowledge and domain-specific competence. Mathematics-related beliefs and self-efficacy, as a dimension of the affective domain are related with students’ performance on geometric problem solving tasks but are not expected to exert a direct predictive effect on the geometrical reasoning (Panaoura, 2014).

The third hypothesis concerns potential differences by grade level and gender across the study variables cognitive reflection, fluid intelligence, mathematical beliefs, mathematical achievement, and performance on geometric solving tasks. The study focuses on adolescence, a period characterized by notable progress in reasoning abilities (Barrouillet et al., 2011; Lemos et al., 2025) and by the gradual shift from concrete to abstract forms of thought within mathematical learning.

Geometry, in particular, represents a domain in which reasoning develops progressively as students acquire greater capacity for abstraction and coordination of representations (Duval, 1998, 2006). Accordingly, we expect that performance on all variables will differ significantly across grade level, with older students outperforming younger ones. Considering gender differences, and in line with prior evidence showing minimal gender differences in mathematics and reasoning (Else-Quest et al., 2010; Hyde et al., 2008; Lindberg et al., 2010), no significant gender differences are anticipated in the measures of geometric problem solving and fluid intelligence. By contrast, cognitive reflection, however, where some studies have reported small differences favoring males (Campitelli and Gerrans, 2014; Frederick, 2005; Primi et al., 2016), hence they are expected in this sample as well.

## 2 Materials and methods

### 2.1 Participants

The final sample comprised 303 students. They were enrolled in Grades 8 to 11 (equivalent to Educación Secundaria Obligatoria and Bachillerato in the Spanish system). The sample included 184 female students (60%) and 119 male students (40%), distributed across grade levels as follows: Grade 8 (*n* = 107; 43 males, 64 females), Grade 9 (*n* = 76; 30 males, 46 females), Grade 10 (*n* = 79; 33 males, 46 females), and Grade 11 (*n* = 41; 13 males, 28 females). In this study, the term “grades” refers to students ‘academic performance in mathematics, whereas “grade level” is used to indicate school year (Grades 8–11).

Participants were recruited from a charter secondary school located in a medium-sized urban area outside Madrid, serving a predominantly middle socioeconomic population They followed the mainstream academic mathematics curriculum corresponding to Grades 8–11 in the Spanish educational system. The final sample was derived from an initial cohort of 413 students after applying predefined exclusion criteria. Specifically, students with special educational needs (such as ADHD, attention difficulties, dyslexia, or other learning-related diagnoses) those enrolled in vocational or applied mathematics tracks, and those who did not complete one or more of the administered tasks were excluded. These criteria were established to ensure greater curricular homogeneity and instructional comparability across participants, particularly with regard to the mathematical knowledge and instructional background required for the geometric reasoning tasks. Excluding students from alternative educational tracks also reduces potential confounding effects related to differential exposure to geometry content and, as a result, the findings should be interpreted within the context of mainstream academic pathways.

### 2.2 Instruments

#### 2.2.1 Geometrical reasoning test

The instrument used to assess students’ geometry performance was the Geometric Reasoning Test (GRT). The test comprises 11 problems covering two-dimensional content (e.g., areas, perimeters, loci, and metric transformations), as well as occasional three-dimensional content (e.g., surface area of polyhedral). The items were systematically organized with increasing complexity, progressing from items requiring the identification of basic geometric elements to problems that demand the coordination of multiple problem-solving strategies (e.g., spatial orientation, pattern recognition, and integration of geometric relations). Across all items, participants are required to transition from an initial intuitive interpretation to more reflective and formal reasoning, integrating visual and discursive information to construct geometric meaning.

The response format was aligned with the cognitive demand of each item. Seven items required open-ended graphical or spatial constructions, three items combined multiple-choice selection with written or graphical justification, and one item used a multiple-choice format without justification. Therefore, although the total GRT score was dichotomous, scoring did not reduce performance to answer selection.

Preliminary psychometric analyses were conducted to examine the internal structure and reliability of the GRT. Internal consistency was modest (Cronbach’s *α* = 0.51). However, this estimate should be interpreted in light of the conceptualization of geometrical reasoning as a multidimensional construct encompassing distinct, though related, processes. Accordingly, the GRT was designed as a brief, heterogeneous problem-solving measure. Its items intentionally cover different geometrical contents and cognitive demands, including perceptual recognition, representational transformation, spatial interpretation, and discursive justification. In such contexts, relatively low internal consistency coefficients are expected and reflect construct heterogeneity rather than measurement deficiency. Moreover, the dichotomous scoring format and the inclusion of cognitively demanding items likely contributed to attenuating reliability estimates. Item–total correlations showed moderate variability, indicating differential contributions of items to the overall construct. Likewise, item difficulty indices spanned a wide range, from highly demanding to moderately accessible items, consistent with the intended progression in task complexity. Therefore, the modest alpha coefficient is interpreted as reflecting the heterogeneity of the construct.

To further explore construct validity, an exploratory factor analysis was conducted using principal axis factoring with Oblimin rotation. Sampling adequacy was acceptable (KMO = 0.63), and Bartlett’s test of sphericity was significant, χ^2^(55) = 195.79, *p* < 0.001. The results supported a multidimensional structure, with factors corresponding to distinct geometrical reasoning demands. This pattern is consistent with the theoretical design of the instrument, which integrates visuospatial processing, geometrical transformations, and higher-order problem-solving. Taken together, these findings provide preliminary evidence supporting the construct validity and scoring reliability of the GRT. The measure is therefore best interpreted as a heterogeneous indicator of geometrical reasoning within problem-solving contexts, rather than as a unidimensional scale. Given the exploratory nature of this first validation, the factor solution should be interpreted as preliminary evidence of the instrument’s internal structure.

The problems were theoretically classified into three categories reflecting distinct cognitive demands. The first category, identification and recognition of geometric elements, includes items involving basic shapes (triangles, quadrilaterals, circles, irregular figures, and polyhedra) and their properties (alignment, perimeters, areas, angles, and loci). These items assess the ability to move beyond perceptual features to identify mathematically relevant properties. The second category, geometrical transformations, comprises both isometries (e.g., symmetries, rotations, and translations) and non-isometric relations (e.g., similarity, congruence, and Pythagorean relationships). These items require coordination across multiple representational registers, in line with Duval’s framework (Duval, 1995a, 1995b, 2006), integrating visual, symbolic, and discursive processes. The third category, modeling and geometric reasoning strategies, includes problems that require the application of algebraic relations, pattern identification, and optimization of distances or paths. These items impose higher demands on strategic coordination and the integration of prior knowledge.

The total GRT score was computed using binary scoring (1 = correct response with appropriate justification; 0 = incorrect or missing response). The scoring procedure focused on the correctness and coherence of the mathematical reasoning displayed in each task. Interrater reliability was assessed using Cohen’s kappa on all responses coded independently by two evaluators, yielding an excellent level of agreement (*κ* = 0.98). Discrepancies (*n* = 2) were resolved through discussion until consensus was reached.

A representative sample of the items is presented below.

##### 2.2.1.1 Hat problem

**Figure 1:**
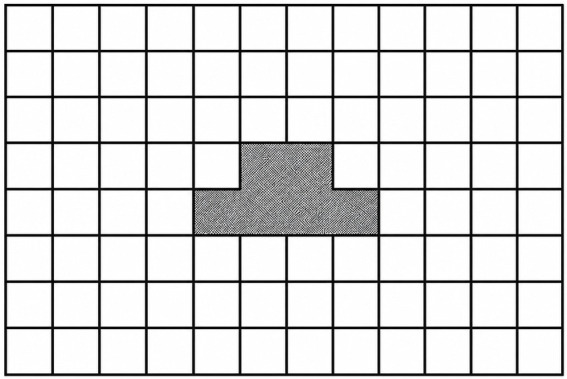
Hat’s problem. Source: Authors’ own elaboration.

In this problem, participants are presented with a grid (see Figure 1) showing the silhouette of a hat, and are asked to select one of the possible solutions or to draw their own proposal if necessary. The item is introduced as follows:

“*In the grid below, there is a figure shaped like a hat. Can you draw a figure that has a greater perimeter than the hat, but a smaller area?*

- *It cannot be done, if it has more perimeter it has to have more area.*
- *It cannot be done, if it has less area it has to have less perimeter.*
- *Yes you can (draw your solution on the grid).*
- *It cannot be concluded”*

This item targets a well-known misconception: that area and perimeter are necessarily related. It examines how students conceptualize this relationship, how this understanding varies with age, and the representational strategies they use. The problem also elicits counterintuitive reasoning. As noted by Stavy and Tirosh (2000), students often struggle to accept relationships that conflict with intuitive expectations, frequently assuming a direct dependence between area and perimeter. Thus, the item provides an ecologically valid context for examining the tension between intuitive and formal reasoning in geometry.

##### 2.2.1.2 Goat problem

**Figure 2:**
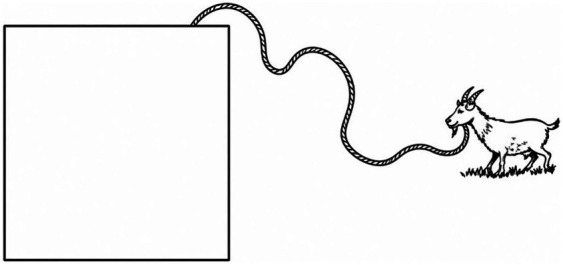
The goat problem. Source: Authors’ own elaboration using ChatGPT, OpenAI, 2025.

This problem involves a goat tied with an 8-m rope to a point on the wall of a 4 × 4 m square plot, and participants are asked to represent the region outside the plot that the goat can reach (see Figure 2). The problem targets concepts of circular arcs and geometric loci, requiring students to identify all points equidistant from a fixed point. It also requires coordinating geometric vocabulary and accurately representing spatial relationships.

#### 2.2.2 Raven’s advanced progressive matrices test

Raven’s Advanced Progressive Matrices (RPMT; Raven et al., 1995) was used as a measure of non-verbal abstract reasoning (fluid intelligence, Gf). The RPMT consists of 60 visual analogy problems. In each item, participants are required to identify the relevant features of an array of abstract figures with one element missing and to select the correct completion from multiple alternatives. The Gf score was the total number of correctly solved items. The reliability of Raven’s Progressive Matrices is well established, supported by their extensive international use and a substantial body of research demonstrating robust psychometric properties, including internal consistency and construct validity (e.g., Carpenter et al., 1990; Raven and Raven, 2003). Previous studies have reported reliability coefficients in the range of *α* ≈ 0.75 or higher.

#### 2.2.3 Cognitive reflection test

A Spanish version of the Cognitive Reflection Test (CRT; Frederick, 2005) was used to assess cognitive reflection. The CRT has become a widely used paradigm to illustrate dual-process accounts of cognition, assessing the ability or disposition to reflect on a question and spontaneously suppress erroneous intuitions that comes to mind (Meyer et al., 2024). An example item is: “A paddle and a ball cost €1.10 in total. The paddle costs €1.00 more than the ball. How much does the ball cost?”. In addition to the three items included in the original test, two additional items proposed by Gómez-Chacón et al. (2014) were incorporated. Participants responded to five open-ended items without time constraints. For each problem, responses were classified into three categories: correct, intuitive (or superficial), and incorrect. The total CRT score was calculated as the number of correct responses. Internal consistency in the present sample was high (Cronbach’s *α* = 0.88).

#### 2.2.4 Mathematics-related beliefs questionnaire

The CreeMat questionnaire is an instrument designed to assess students’ mathematics-related belief systems (Gómez-Chacón et al., 2014). It is grounded in an integrative conceptualization of beliefs, drawing on the Mathematics-Related Beliefs Questionnaire (MRBQ; De Corte et al., 2010; Op’t et al., 2002; Gómez-Chacón et al., 2006). Within this framework, students’ belief systems are defined as subjective conceptions, either implicit or explicit, that are considered to be true and refer to: (1) mathematics as a domain, (2) oneself as a learner of mathematics, and (3) the mathematics classroom context. These beliefs interact with each other and with prior knowledge, shaping students’ engagement in mathematical learning and problem-solving.

The CreeMat questionnaire has been previously used in research settings both in Spain and internationally (Gómez-Chacón et al., 2014; Mello-Román and Gómez-Chacón, 2022). In the present study, the instrument consisted of 11 items (e.g., “I learn mathematics quickly”), rated on a 5-point Likert scale. A total score was computed as an index of overall mathematics-related beliefs, reflecting the degree of endorsement of beliefs related to confidence and perceived competence in mathematics. Internal consistency was estimated using McDonald’s omega (*ω* = 0.75), indicating acceptable reliability. Confirmatory factor analysis supported a two-factor structure: Factor 1 (F1; 4 items; α = 0.65), capturing confidence and students’ perceived mathematical competence (self-efficacy beliefs), and Factor 2 (F2; 7 items; α = 0.70), reflecting broader mathematics-related beliefs. Despite the limited number of items, the reliability coefficients are within acceptable ranges for short scales (Sturmey et al., 2005).

### 2.3 Procedure

Convenience sampling was used in the present study to select participants. Data collection required four sessions of approximately 55 min each conducted across three different days. Each class group completed the Geometric Reasoning Test in the first session (along with two additional short tasks not included in the present study); in a second session, participants completed the CreeMat questionnaire and the Cognitive Reflection Test; the Raven’s Progressive Matrices Test was administered in the third session. The order of the tasks was consistent across the different classroom groups. The tests were administered to students in specific booklets. Data on students’ mathematics achievement (mathematics grades) were obtained from the school’s administrative services, with confidentiality ensured throughout the reporting process. Informed written consent was obtained from parents or legal guardians for all participants, authorizing student participation in the study. Additionally, students were informed on the main study’s objective, the voluntary nature of their involvement, and the confidentiality of their responses. The research was approved by the Ethics Committee of the Universidad Nacional de Educación a Distancia (UNED, Spain).

### 2.4 Data analysis

Data analyses were conducted using IBM SPSS Statistics (version 27) and the PROCESS macro for SPSS. Descriptive statistics (means and standard deviation) were computed for all quantitative variables (Gf, cognitive reflection, mathematics-related beliefs, mathematics achievement, and geometric performance). Normality was evaluated using the Kolmogorov–Smirnov test. Because the assumption of normality was not met for all variables, bivariate associations were examined using Spearman’s rank-order correlation coefficients (rs). Correlation magnitudes were interpreted using conventional criteria (Cohen, 1988) (|0.0| < rs: negligibe; |0.10| ≤ rs < |0.29|: weak; |0.30| ≤ rs < |0.59|: moderate; |0.60| ≤ rs < | 1.00|: strong correlation).

Group differences by gender and grade level were examined for all variables (Gf, CRT, GRT, mathematics grades, CreeMat beliefs, when applicable). Univariate analysis of variance (ANOVAs) were conducted for each dependent variable with gender and grade level entered as fixed factors. When significant main effects were observed, Bonferroni-adjusted *post hoc* comparisons were used to identify specific group differences. In addition, multivariate group differences were evaluated using MANOVA. Assumptions were examined prior to interpretation. Box’s M test was significant, indicating heterogeneity of covariance matrices across groups (Box’s M = 236.14, *p* = 0.001); therefore, Pillai’s Trace was used as the multivariate test statistic due to its greater robustness under violations of covariance homogeneity. Levene’s tests indicated that homogeneity of variance was met for some variables, with moderate violations for others. Nevertheless, given the sample size and the established robustness of ANOVA procedures to moderate departures from normality and homoscedasticity, results were considered appropriate for interpretation.

To examine the unique predictive contribution of the cognitive and non-cognitive variables to geometry, hierarchical multiple regression analyses were conducted. Predictors were entered in theoretically and empirically motivated blocks. Prior to model estimation, regression assumptions were evaluated. Multicollinearity diagnostics indicated no evidence of problematic collinearity among predictors (tolerance values ranging from 0.736 to 0.994; VIF values ranging from 1.006 to 1.359), and indices were within acceptable limits. Standardized residuals further suggested that linearity, normality of residuals, and homoscedasticity were reasonably met. The histogram and normal P–P plot of standardized residuals indicated an approximately normal distribution, while the residuals vs. predicted scatterplot showed no systematic patterns. Standardized residuals were within acceptable ranges.

Finally, to extend the regression framework, mediation and moderation analyses were conducted using PROCESS. Indirect effects were estimated with 5,000 bootstrap resamples and 95% bootstrap confidence intervals. Moderation was tested through interaction terms (e.g., gender and grade level as moderators), including relevant covariates, and effects were interpreted based on the incremental variance explained (ΔR^2^) and the statistical significance of interaction terms.

## 3 Results

### 3.1 Descriptive statistics and group differences

Descriptive statistics (means and standard deviations) for geometry performance, fluid reasoning (Gf), cognitive reflection (CRT), mathematics-related beliefs, and mathematics grades are reported in Table 1. The results indicate both GRT (Grade 8: 1.55; Grade 11: 3.15) and CRT (0.56 to 0.97) scores show an upward trend across grade levels, although the increase is larger for GRT. CRT scores also improve, albeit more gradually. A similar pattern is observed for fluid intelligence (47.64 to 52.52), which rises with age. By contrast, CreeMat scores exhibit a less consistent pattern, with noticeable fluctuations across grade levels. Mathematics achievement increases progressively from Grade 8 to Grade 11(6.91 to 7.93).

**Table 1:** Descriptive statistics (mean and standard deviation), classified by year (8th to 11th, and total) and gender (male-M, female-F and total), variables: GRT (geometry reasoning test), CRT (cognitive refection test), CreeMat (mathematics-related beliefs), math grades.

Grade	Gender	GRT M(SD)	CRT M(SD)	Gf M(SD)	CreeMat M(SD)	Math Grades M(SD)
Year 8	M	1.60 (1.37)	0.79 (1.01)	47.63 (6.14)	35.49 (11.25)	6.70 (1.54)
	F	1.52 (1.17)	0.41 (0.75)	47.65 (5.04)	35.56 (8.62)	7.05 (1.90)
	Total	1.55 (1.25)	0.56 (0.88)	47.64 (5.47)	35.53 (9.71)	6.91 (1.76)
Year 9	M	1.53 (1.50)	1.03 (1.25)	47.00 (6.50)	37.30 (13.79)	7.40 (1.54)
	F	2.00 (1.49)	0.52 (0.78)	47.74 (6.07)	36.74 (11.22)	8.13 (1.28)
	Total	1.82 (1.50)	0.72 (1.01)	47.46 (6.19)	36.96 (12.21)	7.84 (1.42)
Year 10	M	2.55 (1.79)	1.52 (1.58)	48.54 (5.25)	29.76 (16.62)	6.79 (1.43)
	F	2.89 (1.84)	0.83 (1.30)	49.22 (4.71)	33.02 (16.76)	7.85 (1.76)
	Total	2.75 (1.81)	1.11 (1.46)	48.96 (4.89)	31.66 (16.68)	7.40 (1.70)
Year 11	M	3.77 (2.09)	0.85 (0.90)	53.44 (2.79)	29.92 (21.08)	8.23 (1.64)
	F	2.86 (1.88)	0.50 (0.64)	52.00 (3.18)	33.75 (17.21)	7.78 (1.65)
	Total	3.15 (1.97)	0.61 (0.74)	52.52 (3.07)	32.54 (18.35)	7.93 (1.64)
Total	M	2.08 (1.75)	1.06 (1.26)	48.26 (5.99)	33.75 (14.91)	7.07 (1.58)
	F	2.18 (1.65)	0.55 (0.92)	48.50 (5.22)	34.95 (13.09)	7.63 (1.73)
	Total	2.15 (1.69)	0.75 (1.10)	48.41 (5.52)	34.48 (13.82)	7.41 (1.69)

To examine potential differences by gender and grade level (Hypothesis 3), a multivariate analysis of variance (MANOVA) was conducted on geometry performance (GRT), cognitive reflection (CRT), fluid intelligence (Gf), mathematics grades, and mathematics-related beliefs. The results showed a significant effect of grade level, Pillai’s Trace = 0.289, *F*(18, 672) = 3.98, *p* < 0.001, partial *η*^2^ = 0.096, indicating that the outcomes differed across grade levels. A significant multivariate effect of gender was also observed, Pillai’s Trace = 0.230, *F*(6, 222) = 11.07, *p* < 0.001, partial *η*^2^ = 0.230. The grade by gender interaction was not significant, Pillai’s Trace = 0.087, *F*(18, 672) = 1.12, *p* = 0.330, partial *η*^2^ = 0.029, suggesting that gender differences were broadly comparable across grade levels.

Follow-up univariate ANOVAs indicated significant grade level differences in geometry performance (GRT), *F*(3, 227) = 10.04, *p* < 0.001, partial *η*^2^ = 0.117, fluid intelligence (Gf), *F*(3, 227) = 6.41, *p* < 0.001, partial *η*^2^ = 0.078, and mathematics grades, *F*(3, 227) = 5.52, *p* = 0.001, partial *η*^2^ = 0.068. No significant grade level differences were found for CRT (*p* = 0.070), mathematics-related beliefs (*p* = 0.170), or mathematics self-efficacy (*p* = 0.143). Furthermore, Bonferroni-adjusted *post hoc* comparisons were conducted. For GRT, students in Grade 11 scored higher than those in Grade 8 (*p* = 0.002), and students in Grade 10 outperformed those in Grade 8 (*p* < 0.001) and Grade 9 (*p* = 0.027). For Gf, students in Grade 11 scored higher than those in Grades 8 and 9 (both *p* < 0.001) and Grade 10 (*p* = 0.042). For mathematics grades, students in Grade 9 scored higher than those in Grade 8 (*p* < 0.001), and students in Grade 10 obtained higher grades than those in Grade 8 (*p* = 0.031).

With respect to gender, univariate tests showed significant differences in CRT performance, *F*(1, 227) = 33.39, *p* < 0.001, partial *η*^2^ = 0.128, and in mathematics self-beliefs, *F*(1, 227) = 6.47, *p* = 0.012, partial *η*^2^ = 0.028. No significant gender differences were observed for GRT, *F*(1, 227) = 0.181, *p* = 0.671, partial *η*^2^ = 0.001, indicating a negligible effect size; for Gf, with *F*(1, 227) < 0.001, *p* = 0.998, partial *η*^2^ < 0.001; mathematics grades, *F*(1, 227) = 3.97, *p* = 0.05, partial *η*^2^ = 0.017; or overall mathematics-related beliefs, *F*(1, 227) = 0.79, *p* = 0.374, partial *η*^2^ = 0.003. The Gender × Grade interaction was also non-significant, *F*(3, 227) = 0.323, *p* = 0.809, partial *η*^2^ = 0.004. Notably, the latter result refers to the total CreeMat score, whereas the significant gender effect concerned only the self-efficacy factor (Factor 1) of the instrument. No significant interaction effects were observed in any of the univariate analyses. In addition, a sensitivity power analysis further indicated that the available gender-group sample sizes (119 boys and 184 girls) provided approximately 80% power to detect gender differences of *d* = 0.33 or larger at *α* = 0.05. Thus, the non-significant gender effect on GRT should be interpreted as indicating no evidence of small-to-moderate gender differences in this sample, while very small effects cannot be ruled out.

### 3.2 Interrelationships among variables

Correlations among geometry performance, Gf, CRT, mathematics-related beliefs, and school mathematics grades are presented in Table 2. The pattern of interrelationships was consistent with the expected associations (Hypothesis 1), supporting the inclusion of these predictors in the subsequent regression analyses. As shown in Table 2, all variables were significantly and positively correlated (*r*s = 0.18–0.5, *p*s < 0.01). In particular, geometric performance showed moderate association with fluid intelligence and mathematics achievement. More specifically, fluid intelligence was positively associated with mathematics achievement, and cognitive reflection was related to mathematics-related beliefs. In addition, geometric performance showed a close relationship with fluid intelligence, while higher mathematics achievement was linked to more positive mathematics related beliefs. It should be noted that, although all correlations were positive, their magnitude varied, as some associations were relatively modest yet significant (e.g., self-efficacy beliefs and GRT: *r* = 0.16, *p* < 0.05). Overall, the results indicate that cognitive variables (Gf, GRT, CRT), mathematics achievement and beliefs are interrelated and may jointly contribute to the patterns observed across grade levels.

**Table 2:** Spearman bilateral correlations between geometry reasoning test (GRT), cognitive reflection test (CRT), non-verbal reasoning (Gf), mathematics grades, mathematics beliefs (CReeMat), and self-efficacy beliefs.

	1	2	3	4	5	6
1. GRT	1					
2. CRT	0.29**	1				
3. Gf	0.37**	0.23**	1			
4. Math grades	0.28**	0.18**	0.39**	1		
5. CreeMat (total math beliefs)	0.20**	0.37**	0.30**	0.50**	1	
6. Self-efficacy beliefs	0.16*	0.22**	0.23**	0.38**	0.91**	1

** The correlation is significant at the 0.01 level, * the correlation is significant at the 0.05 level (two-tailed).

### 3.3 Predicting geometry performance: hierarchical regression analyses

To test Hypothesis 2 and identify the unique predictors of students’ geometric performance (GRT), two hierarchical multiple regression analyses were conducted. Both models followed the same theoretically driven sequence of entry, also consistent with the correlational pattern of results. The two regressions differed only in the final step: first, the total score of mathematics-related beliefs (CreeMat) was added to test whether beliefs explained additional variance beyond cognitive and achievement variables; second, the final step included mathematics self-beliefs (CreeMat F1) instead of total score. These results can be seen in Tables 3, 4.

**Table 3:** Summary of hierarchical regression models predicting performance on geometric problem-solving tasks considering the total score of total beliefs about mathematics (CreeMat).

Model	Adjusted R^2^	*F*	*df*	*p*
1: Year	0.02	5.9	(1,233)	0.016
2: +Gf	0.16	22.69	(1,232)	<0.001
3: +CRT	0.19	19.65	(1,231)	<0.001
4: + Math grades	0.225	17.95	(1,230)	<0.001
5: CreeMat (total beliefs)	0.222	14.37	(1,229)	<0.001

**Table 4:** Summary of hierarchical regression models predicting performance on geometric problem- solving tasks considering the total score self-efficacy beliefs.

Model	Adjusted R^2^	*F*	*df*	*p*
1: Year	0.02	5.9	(1.233)	0.016
2: +Gf	0.16	22.69	(1.232)	<0.001
3: +CRT	0.19	19.65	(1.231)	<0.001
4: + Math grades	0.225	17.95	(1.230)	<0.001
5: Self-efficacy beliefs	0.223	14.41	(1.229)	<0.001

Specifically, grade level was entered first to control for developmental differences; fluid intelligence (Gf) was entered next as a domain-general cognitive variable and accounted for 16% of the variance, *R^2^* = 0.16, *F*(2, 234) = 22.69, *p* < 0.001; cognitive reflection (CRT) was added subsequently as a more specific reasoning-related ability, adding the cognitive variable of cognitive reflection increased the explained variance to 19%, *F*(3, 234) = 17.95, *p* < 0.001; and school mathematics grades were entered as an indicator of prior academic achievement, and increased the explained variance to 22.5% with *F*(4, 234) = 17.94, *p* < 0.001. The subsequent inclusion of mathematics-related beliefs did not meaningfully improve the model, with the final model explaining 22.3% of the variance, *F*(5,234) = 14.37, *p* < 0.001. Therefore, the addition of the variable of beliefs about mathematics did not result in a significant increase in the amount of variance explained in geometric performance (GRT) (*ΔR*^2^ < 0.01, *p* > 0.05).

The final model accounted for a moderate but meaningful proportion of variance in geometry performance, 22%, where fluid intelligence (*β* = 0.259, *p* < 0.001), cognitive reflection (β = 0.206, *p* < 0.001), and mathematics grades (β = 0.215, *p* = 0.002) emerged as significant unique predictors of geometry performance. Mathematics-related beliefs, however, were not significant (β = −0.035, *p* = 0.593), and did not increase the explained variance of the model (*ΔR*^2^ < 0.01, *p* > 0.05). Multicollinearity diagnostics showed no problematic overlap among predictors, with VIF values ranging from 1.064 to 1.359. Thus, cognitive reflection contributed uniquely to geometry performance beyond fluid intelligence and prior mathematics achievement.

A parallel model was examined including Factor 1 score from the CreeMat cuestionnaire (instead of the total CreeMat score). The results showed the same pattern: beliefs did not contribute additional explanatory variance beyond grade level, fluid intelligence, cognitive reflection, and mathematics grades. In this model, the total variance explained remained virtually unchanged (*R*^2^ = 0.223), *F*(5, 234) = 14.41, *p* < 0.001. As no meaningful increase in explained variance was observed, the previous model was retained as the most parsimonious solution. The direct effects of fluid intelligence, cognitive reflection, and mathematics grades remained consistent with those reported in the previous model.

Building on the hierarchical regression results, to further examine the relationships among variables beyond the primary regression analyses, we conducted mediation and moderation analyses. We next report the mediation analyses (beliefs as a mediator, controlling for gender and grade level), followed by moderation analyses examining whether the Gf–geometry and CRT–geometry performance associations vary by gender or grade level.

### 3.4 Mediation analyses: mathematics-related beliefs as a mediator

**Figure 3:**
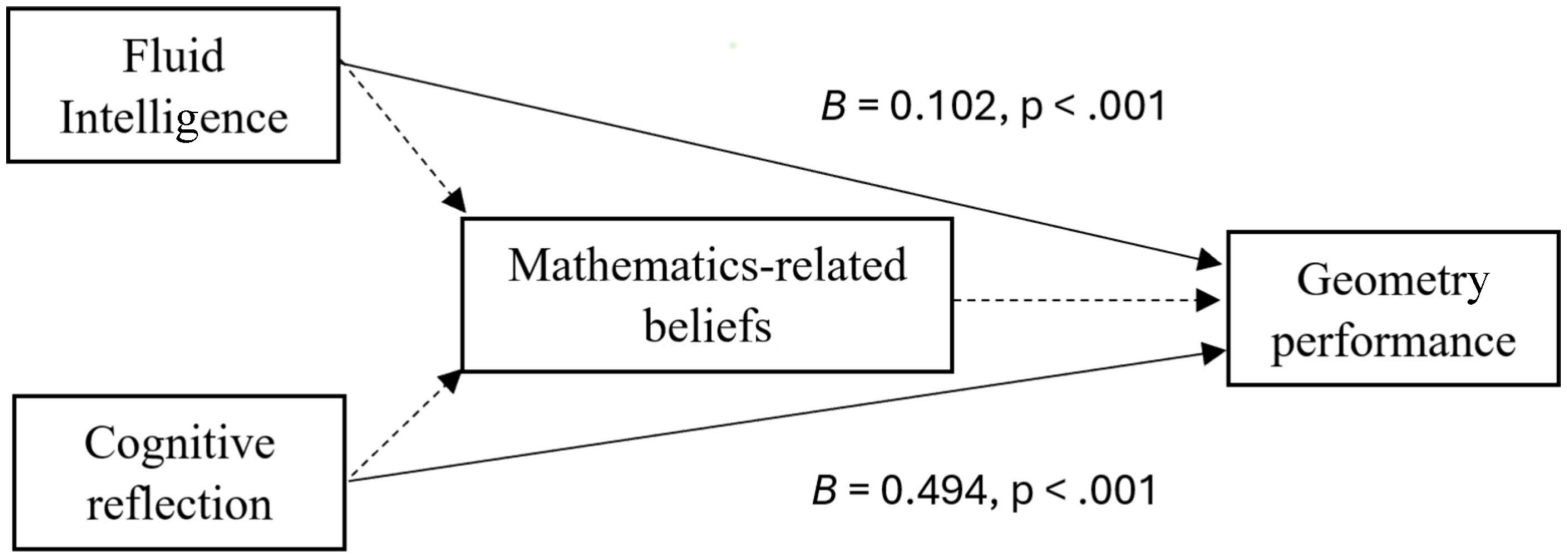
Model of the relationships tested among Gf, cognitive reflection, mathematics-related beliefs, and geometry performance. Covariates were gender and grade/course. Solid lines represent significant direct effects. Dashed lines represent mediation paths that were tested but not supported. Moderation effects were tested but not supported in this sample.

To examine the potential indirect role of mathematics-related beliefs, as assessed by the CreeMat questionnaire, mediation analyses were conducted. CreeMat scores were used as an index of students’ belief systems. These analyses aimed to determine whether beliefs account for the associations between cognitive variables (fluid intelligence and cognitive reflection) and geometry performance, controlling for gender and grade level. Two mediation models were tested, one with Gf as the predictor and one with CRT as the predictor, controlling for gender and grade/course. Indirect effects were estimated using 5,000 bootstrap resamples and 95% confidence intervals. To facilitate the interpretation of the extended analyses, Figure 3 summarizes the conceptual model tested in this study, including the direct effects of fluid intelligence (Gf) and cognitive reflection (CRT) on geometry performance, the indirect pathway via mathematics-related beliefs, and the effects tested across gender and grade level.

In the model including Gf, the direct effect of Gf on geometry performance was significant, *B* = 0.1022, *SE* = 0.0170, *t* = 5.85, *p* < 0.001, 95% CI [0.0678, 0.1360]. However, the indirect effect via mathematics-related beliefs was not significant, *B* = 0.0043, *SE* = 0.0048, 95% CI [−0.0027, 0.0159]. Similarly, in the model with CRT as the predictor, the direct effect on geometry performance was significant, *B* = 0.4940, *SE* = 0.0934, *t* = 5.30, *p* < 0.001, 95% CI [0.3117, 0.6793], whereas the indirect effect via mathematics-related beliefs was not significant, *B* = −0.0125, *SE* = 0.0389, 95% CI [−0.0898, 0.0632]. These results did not provide evidence that mathematics-related beliefs mediate the associations between cognitive variables, Gf or CRT, and geometry performance in this sample. This pattern suggests that the associations between cognitive variables and geometry performance are direct rather than mediated by belief systems.

### 3.5 Moderation analyses: grade level and gender as moderators

We next examined whether the associations between cognitive predictors and geometry performance varied by grade level or gender. In each model, the remaining grouping variable was included as a covariate. For Gf, the Gf × grade level interaction was not statistically significant, *ΔR*^2^ = 0.0026, *F*(1, 230) = 0.7079, *p* = 0.40. The Gf × gender interaction was also not statistically significant, *ΔR^2^* = 0.0035*, F*(1, 230) = 0.9690, *p* = 0.32. For CRT, the CRT × gender interaction was not statistically significant, Δ*R*^2^ = 0.0003*, F*(1, 298) = 0.1075, *p* = 0.7433. The CRT × grade level interaction did not reach conventional levels of statistical significance, *ΔR*^2^ = 0.0089, *F*(1, 298) = 2.96, *p* = 0.085. Overall, the moderation analyses indicated that interaction effects were small and not statistically reliable, suggesting that the predictive relations between the cognitive predictors (Gf and CRT) and geometry performance were broadly similar across grade and gender in our sample.

## 4 Discussion

The present study examined the contribution of cognitive reflection (CRT), fluid reasoning (Gf) and mathematics achievement to geometry performance in adolescents and further examined whether mathematics-related beliefs mediate these associations and whether these associations differ as a function of grade level or gender.

Considering the first and second hypotheses, the relationship among all cognitive and affective variables (belief systems) showed significant positive correlations with geometric performance, as predicted. These findings are consistent with recent studies that highlight the joint contribution of cognitive and affective factors to mathematical performance (Semeraro et al., 2020; Zuo et al., 2024). Although mathematics-related beliefs (CreeMat) also correlated significantly with all other variables, most notably with mathematics grades, their role in predicting performance on geometric performance appeared to be peripheral, as suggested by the results of the subsequent regression analysis This is consistent with other studies (Gómez-Chacón et al., 2014; Panaoura, 2014; Semeraro et al., 2020) in particular with Zuo et al. (2024) who concluded that although affective-motivational factors are important for learning, their predictive value diminishes when cognitive variables are considered concurrently. In light of these findings, the results of the present study suggest that mathematics-related beliefs operate more as contextual enablers than as direct predictors of performance on geometrical solving tasks.

The hierarchical regression analyses confirmed the predictive contribution of cognitive reflection, fluid intelligence, and academic achievement to performance on geometric problem-solving tasks. Including mathematics-related beliefs in the final model did not show a significant increase in explained variance, suggesting the greater relevance of cognitive factors in the reasoning process. These findings are in line with prior research that highlights the influence of fluid intelligence on both mathematical and geometric performance (Giofrè et al., 2014; Mammarella et al., 2017; Primi et al., 2013; Roth et al., 2015) and the studies that show that mathematical performance is supported by multiple cognitive skills (Amland et al., 2025). Furthermore, they support dual-process theories of reasoning, that sustain that cognitive reflection enables individuals to override intuitive responses (System 1) in favor of more deliberate, effortful reasoning (System 2) (Evans and Stanovich, 2013; Frederick, 2005; Kahneman and Frederick, 2002). In this framework, the CRT captures key components of students’ capacity for analytical and self-regulated thinking skills, that are essential for solving non-routine geometric problems.

This result suggests that geometric problem-solving in adolescence is supported not only by prior mathematical knowledge, but also by domain-general skills that allow students to inhibit misleading visual or intuitive responses and to reorganize the information given in the task. Thus, the role of CRT in the present study should not be interpreted as a simple proxy for mathematical skill, but as an indicator of students’ tendency to resist or revise intuitive responses in situations requiring deliberate reasoning. In this regard, Meyer et al. (2024) provide relevant construct-validity evidence by showing that CRT performance reflects more than mathematical aptitude: its distinctive contribution lies in the capacity to resist or revise intuitive but misleading responses. Applied to the present findings, this supports the interpretation that cognitive reflection may help students move beyond an immediate perceptual reading of geometric figures toward the analysis of underlying geometrical relations. Thus, although CRT and Gf may share variance as domain-general cognitive skills, the absence of problematic multicollinearity suggests that the regression results are compatible with a complementary interpretation: Gf may support abstract, logical, and non-verbal reasoning, whereas CRT may be more closely related to resisting initially plausible but potentially misleading responses. Studies such as that of Dolz et al. (2026) have captured the interaction between CR and Gf in relation to conditional reasoning and use of logical language. A similar exploration would be open for our study.

The amount of explained variance in the present regression model should be interpreted as moderate but meaningful, particularly considering that the predictors combined domain-general cognitive skills and prior academic achievement rather than task-specific instructional variables. Direct comparisons with previous studies should be made cautiously, since geometry performance is multidimensional construct and different models emphasize different predictors. For example, Weckbacher and Okamoto (2018) reported that regarding spatial visualization explained approximately 7% of the variance in high-school students’ geometry performance; adding mental rotation increased the explained variance to approximately 10%, although this increment was not statistically significant. In this sense, the approximately 22% of variance explained in the present study suggests that fluid intelligence, cognitive reflection, and prior mathematics achievement provides a broader, though still partial, predictive account of adolescents’ geometric problem-solving.

The mediation analyses showed consistent direct effects of both CRT and Gf on geometry performance even after controlling for gender and grade. In contrast, the bootstrapped indirect effects via mathematics-related beliefs were not supported. This pattern suggests that, in this sample, mathematics-related beliefs—as operationalized here—do not explain why higher cognitive ability or greater cognitive reflection is associated with better geometry performance. The non-significant indirect effects suggest that, in this cohort, broad mathematics-related beliefs did not constitute the intervening route linking Gf or CRT to geometry performance. This coincides with results that have indicated that beliefs are not always stable or show weaker or indirect relationships (Panaoura, 2014). Self-efficacy often predicts achievement, but findings are inconsistent across interventions and contexts, some studies show weaker or indirect relationships (Zakariya, 2022).

Several interpretations may account for this absence of mediation. First, mathematics-related beliefs may operate as a parallel determinant of geometry performance rather than as an intervening mechanism linking cognitive predictors to geometry outcomes. Second, mediation tests are sensitive to construct specificity and measurement precision, and it should be possible that if beliefs capture broad mathematics self-beliefs rather than geometry-specific competence expectations, the indirect pathway may be attenuated. Third, given the cross-sectional nature of the data, temporal precedence cannot be established and, hence, longitudinal designs are needed to evaluate whether changes in cognitive predictors precede changes in beliefs, which in turn predict growth in geometry performance.

Importantly, the absence of mediation should not be read as evidence that mathematics-related beliefs are irrelevant. Indeed, geometry-specific research has linked students’ self-efficacy beliefs about using representations with their cognitive performance in geometrical tasks (Panaoura, 2014), and recent evidence from Spanish secondary students shows that mathematics self-efficacy is a significant predictor of mathematical competence (Ortega-Rodríguez, 2025). Rather, it indicates that, in this cross-sectional design and with broad mathematics-related beliefs, these beliefs did not explain the pathway from cognitive predictors to geometry performance. This interpretation is consistent with recent studies emphasizing that mathematics self-efficacy and related belief constructs are multidimensional and dynamic, and that their relationship with performance cannot be reduced to a single explanatory pathway: depending on the theoretical model, mathematical content, task demands, and measurement design, these constructs may operate as predictors, mediators, moderators, or outcomes in reciprocal relations (Street et al., 2024). Therefore, the non-significant indirect effects observed here suggest not that beliefs are unimportant, but that broad mathematics-related beliefs, including self-efficacy beliefs, did not account for the association between CRT, Gf and performance on the GRT.

The moderation analyses did not indicate significant interactions between Gf/CRT and grade or gender in predicting geometry performance. All interaction terms were non-significant, with only a small non-significant increment in explained variance for the CRT × Grade interaction. In other words, within the range of grade levels and the gender composition of the present sample, the cognitive predictors’ relations with geometry performance appear consistent across groups. At the same time, this invariance should be interpreted carefully. The absence of significant interactions suggests that grade level and gender did not substantially alter the strength of the cognitive–geometry relations in this sample, but it does not imply that developmental or gender-related differences are absent in other dimensions of mathematical learning and reasoning. In sum, the extended analyses indicate that cognitive predictors show robust direct links to geometry performance, with limited evidence that these relations operate through mathematics-related beliefs or differ meaningfully by grade or gender in this adolescent sample. Also, at the mean level, the results revealed selective group differences: geometry performance and Gf differed across grade level, whereas CRT and mathematics-related beliefs did not; in addition, CRT differed by gender.

On one hand, Grade level differences also emerged in mathematics-related overall beliefs. This pattern is consistent with the characteristics of the sample: from Grade 9 onwards, students had opted for the academic track in mathematics, as opposed to vocational or applied mathematics within the Spanish curriculum. Although variability within grade level indicates heterogeneous tendencies, older students in this sample not only study mathematics but have deliberately chosen to continue pursuing it. In addition, as mathematical beliefs tend to crystallize with age, this helps explain the higher CreeMat scores of older students (Grade 11) compared with younger ones (Grade 8).

On the other hand, gender did not significantly affect performance on geometry solving tasks, fluid intelligence, mathematics achievement or beliefs about mathematics, including self-efficacy. This suggests that the gender gap sometimes reported in STEM fields should not be attributed straightforwardly to inherent differences in individual capabilities or in the cognitive variables underlying mathematical and geometrical reasoning. Our findings are consistent with recent meta-analytic and empirical evidence suggesting that gender differences in mathematics performance are negligible or very small in many educational contexts (e.g., Brown and Alexandersen, 2020; Rodríguez et al., 2020) or with studies in Spanish context (Ramírez-Uclés and Ramírez-Uclés, 2020). Some of these studies point out the importance of exploring the role of the type of task, the educational context and variability within the group (Becker and Hall, 2024).

However, a small advantage for boys was observed in the cognitive reflection test (CRT). This is consistent with previous reports (Campitelli and Gerrans, 2014; Frederick, 2005; Primi et al., 2016) and with recent meta-analytic evidence showing that males tend to score higher than females in cognitive reflection, although the magnitude of this difference is small and partly depends on the type of CRT used, particularly its numerical format (Otero et al., 2024). So, the gender difference observed in CRT should be interpreted cautiously, although this does not undermine the predictive role of CRT observed in the regression analyses; rather, it indicates that CRT was relevant for explaining individual differences in geometry performance, but not for producing a gender-based advantage in the GRT.

Thus, the results highlight a dissociation between mean-level differences and predictive relations: adolescents may differ in average levels of performance and specific cognitive characteristics across grades and by gender for CRT, yet the strength of the cognitive–geometry associations appears broadly stable across these groups. These findings are especially relevant in adolescence, a period marked by increasing abstraction and representational coordination in mathematical learning (Barrouillet et al., 2011), and influenced by both developmental changes in cognitive performance and increasing differentiation according to curricular content and cognitive demands (Lemos et al., 2025). However, because the present study used a cross-sectional design, these grade-level differences should be interpreted as age-related differences across school years, rather than as longitudinal evidence of developmental change within the same students.

The GRT highlighted the transformations and conversions of representations across semiotic registers, enabling the evaluation of students’ progression from immediate visual recognition to more advanced reasoning involving abstraction and formal justification. These findings are consistent with dual-process theories of reasoning, as geometry requires balancing figural intuition with discursive control (Schoenfeld, 1985; Kuzniak et al., 2016). Using Duval’s framework, this coordination of semiotic registers becomes especially important to identify the geometric reasoning during adolescence, when students are expected to develop more abstract and formal reasoning.

Overall, these results illustrate the associations among general cognitive abilities and academic experience in shaping students’ performance on geometric problem-solving. While fluid intelligence provides a foundation for abstraction and non-verbal reasoning, cognitive reflection appears to facilitate inhibition of intuitive responses and construction of structured arguments. Academic achievement, in turn, reflects the cumulative knowledge base that supports this process, while beliefs influence weaker or indirect relationships in how these cognitive skills are mobilized and sustained.

The study’s main limitation lies in the restricted selection of variables. Although the focus was placed on key cognitive and affective aspects, other relevant factors (such as metacognitive strategies, self-regulated learning, or emotional factors like mathematics anxiety) were not considered and should be explored in future studies. In our study the consideration regarding beliefs system allows to go deeper on the self-concept beliefs and self-efficacy beliefs in upcoming studies. Additionally, it should be noted that inclusion criteria necessarily limit the generalizability of the results, and the present findings should be interpreted within the context of mainstream academic pathways. In particular, the findings should not be extrapolated to students in vocational or applied mathematics tracks or to those with special educational needs, whose instructional trajectories may differ substantially. Further research focused specifically on students with special educational needs and alternative educational tracks would be valuable to better understand the potentially distinct cognitive and educational factors involved in geometric reasoning across diverse learning contexts. The results have opened a line of deepening with respect to the instrument of Geometric Reasoning Test (GRT) used in two directions: (1) in the structure and presentation of geometric tasks and (2) in the analysis of categories to discriminate types of semiotic representations. We agree with Gagatsis et al. (2022) that the structure and presentation of geometric tasks can influence students’ representational strategies and creative engagement. This supports the idea that task design in the GRT, which integrates multiple representational challenges, plays a crucial role in eliciting students’ discursive and sequential apprehensions according to Duval framework.

Despite these limitations, the present work contributes to a growing body of literature that positions geometry as a privileged domain for exploring how learners construct meaning through reasoning and representational activity (Castro et al., 2022; Kuzniak et al., 2016) and the predictive contribution of all variables: cognitive reflection, fluid intelligence, mathematical achievement, beliefs about mathematics and self-efficacy beliefs on the students’ performance on geometric problem-solving tasks.

The educational implications are twofold. First, the findings underscore the importance of assessing and supporting cognitive reflection and reasoning in school geometry. Second, they reinforce the need to develop didactic strategies that activate reflective processing and help students shift from intuitive to analytical reasoning. These strategies may be particularly valuable in geometry, where understanding often depends on students’ ability to coordinate visual, discursive, and symbolic representations to solve complex problems. In this regard, previous studies have emphasized the role of representational flexibility and the coordination of multiple semiotic registers as essential components of meaningful geometric activity (Deliyianni et al., 2015; Gagatsis et al., 2022; Michael-Chrysanthou et al., 2024). Promoting students’ capacity to move between representations and adopt flexible problem-solving strategies can help bridge intuitive and formal reasoning, fostering deeper mathematical understanding.

## 5 Conclusion

This study examined adolescents’ performance on geometric problem-solving tasks by considering both dual-process theories of reasoning and Duval’s theory of semiotic representations. The findings provide evidence that students’ performance on geometry solving tasks is shaped by the combined influence of cognitive abilities, beliefs about mathematics (including self-efficacy) and accumulated academic experience.

Across adolescence, geometric reasoning shows differences in the perception and coordination of representations. Cognitive reflection and fluid intelligence were identified as significant predictors of geometric problem-solving performance, highlighting the role of analytical reasoning in geometrical problem solving. Mathematics-related beliefs were positively associated with performance but showed weaker associations when examined alongside cognitive and achievement-related variables. The final predictive model was statistically significant and indicated that Gf, CRT, and mathematics grades accounted for a meaningful proportion of variance in geometric problem-solving, whereas mathematics-related beliefs and self-efficacy did not explain additional variance once cognitive and achievement-related variables were included.

In short, the findings highlight the relevance of cognitive reflection and representational flexibility on geometry, contributing to a clearer understanding of how students solve geometric problems during secondary education. Overall, these results suggest that adolescents’ geometric problem-solving depends primarily on reflective cognitive skills, prior mathematical experience, and the coordination of semiotic representations, with mathematics-related beliefs playing a broader contextual role.

## Data Availability

The raw data supporting the conclusions of this article will be made available by the authors, without undue reservation.
